# Nondestructive, fatigue cyclic, and ramped cyclic biomechanical testing of surgical techniques for stabilization of the lumbosacral junction in dogs

**DOI:** 10.1111/vsu.70042

**Published:** 2025-11-14

**Authors:** Lucas A. Smolders, Raphael Arz, Frank Steffen, Antonio Pozzi, Brian Park

**Affiliations:** ^1^ Clinic for Small Animal Surgery, Vetsuisse Faculty Zurich Switzerland; ^2^ IVC Evidensia Bessy's Small Animal Clinic Watt Switzerland

## Abstract

**Objective:**

To compare transarticular screw fixation (TSF), pedicle screw‐rod fixation (PSRF), and screws and polymethylmethacrylate (SPMMA) for stabilization of the canine lumbosacral junction (LSJ).

**Study design:**

Ex vivo biomechanical study.

**Sample population:**

Twenty‐one L7‐S1 canine spine specimens collected from adult, large‐breed dogs.

**Methods:**

Specimens were subjected to nondestructive biomechanical testing in flexion/extension (FE), lateral bending (LB), and axial rotation (AR). Subsequently, the L7‐S1 joint was destabilized by dorsal laminectomy and partial discectomy of L7‐S1 and then stabilized by (1) TSF, (2) PSRF, or (3) SPMMA (*n* = 7 specimens/group). Stabilized specimens were subjected to nondestructive biomechanical testing, fatigue cyclic testing, and ramped cyclic testing. For nondestructive and fatigue cyclic testing, range of motion (ROM) was calculated for each testing condition/stabilization method, while for ramped cyclic testing the bending moment necessary to reach a ROM of 5.0°, 7.5° and 10.0° and failure mode were recorded. Linear mixed models and Fisher's exact test were used to analyze continuous parameters and failure modes, respectively.

**Results:**

All stabilization methods resulted in an equal reduction in ROM in FE. Fatigue cyclic testing resulted in minor increases in ROM compared to baseline for all groups. Ramped cyclic testing resulted in failure of all groups, with no differences between groups for the bending moment necessary to reach 5.0°/7.5°/10.0° of ROM. The main failure mode for each method was progressive screw loosening.

**Conclusion:**

Transarticular screw fixation, PSRF and SPMMA provided immediate biomechanical stability to the LSJ and were similar when subjected to fatigue and ramped cyclic testing.

**Clinical significance:**

Transarticular screw fixation, PSRF, and SPMMA are biomechanically effective options for stabilizing the canine LSJ. This study supports clinical use of these procedures.

**Abbreviations**
AR: Axial rotation
DLSS: Degenerative lumbosacral stenosis
FE: Flexion/extension
LB: Lateral bending
LS: lumbosacral
LSJ: lumbosacral junction
NZ: Neutral zone
NZS: Neutral zone stiffness
PSRF: Pedicle screw‐rod fixation
ROM: Range of motion
SPMMA: screws and polymethylmethacrylate
TSF: Transarticular screw fixation

## 1 INTRODUCTION

Degenerative lumbosacral stenosis (DLSS) is a common cause of lumbosacral (LS) disease in dogs.^1^, ^2^ Degenerative changes of different spinal components may cause compression of the cauda equina nerve roots with consequent low back pain, lameness, and neurological deficits.^1^, ^2^ Surgical treatment of DLSS commonly consists of a decompressive procedure, involving a dorsal laminectomy, partial discectomy and/or foraminotomy in case of foraminal stenosis.^3^, ^4^ Additional stabilization after decompressive surgery may be advocated, with the goals of promoting LS fusion and eliminating pain caused by motion and dynamic compression of the cauda equina nerve roots.^4^, ^5^, ^6^

Various methods for surgical stabilization of the lumbosacral junction (LSJ) have been described.^2^, ^5^, ^7^, ^8^, ^9^, ^10^, ^11^, ^12^ On the basis of a recent questionnaire among veterinary neurologists and surgeons (unpublished data), the three most common methods applied to stabilize the LSJ are (1) transarticular screw fixation (TSF) using cortical screws placed through the L7‐S1 articular facet into the body of S1;^8^, ^10^ (2) pedicle screw‐rod fixation (PSRF) using (polyaxial) pedicle screws placed into the pedicles of L7 and S1 and interconnected with rods fixed in the screw heads,^5^, ^12^ and (3) locking head screws placed into the pedicles of L7 and S1, combined with transarticular cortical screws and interconnected with polymethylmethacrylate (SPMMA).^9^

The main purpose of these stabilizing procedures is to provide rigid short‐ and long‐term stabilization of the LSJ. Although the procedures described above are commonly applied in clinical cases, there is a clear lack of biomechanical data with respect to immediate postoperative stability, the resistance to fatigue testing, and their mode of failure.

The objective of this study was therefore to assess and compare these three methods for LS stabilization with respect to direct postoperative stability, stability after continuous fatigue cyclic testing and ramped fatigue testing, and failure modes. It was hypothesized that (1) all three stabilization methods would provide direct postoperative rigid fixation of the LSJ; (2) PSRF and SPMMA would be superior to TSF for withstanding continuous and ramped fatigue cyclic testing, and (3) the main mode of failure for TSF would be screw loosening and pullout, whereas for PSRF and SPMMA the main mode of failure would be fracture of the sacrum.

## 2 MATERIAL AND METHODS

Twenty‐one L6‐S3 canine spine specimens isolated from 21 normal dogs were used. All dogs were client‐owned and euthanized due to reasons unrelated to spinal disease. Owner consent for using the canine cadavers for scientific purposes was obtained for all dogs by way of a signed owner consent form. The median age of the dogs was 6 years (range: 1–13 years) and the median weight was 33.2 kg (range: 27.0–48.0 kg; Table 1). Dorsoventral and lateral radiographs were taken to exclude spinal specimens demonstrating anomalies or signs of severe degenerative pathology. After collection, specimens were wrapped in saline‐soaked towels and frozen at −20°C.

**TABLE 1:** Overview of the canine cadaveric spines used for the study.

Specimen	Breed	Age	Sex	Weight (kg)	Test group
1	Appenzeller–husky cross	13 years 2 months	M	25.2	TSF
2	Mongrel	2 years 8 months	FN	24.9	TSF
3	Boxer	6 years 7 months	FN	35.0	TSF
4	German shepherd	7 years	M	45.2	TSF
5	Labrador retriever	13 years 2 months	MN	33.2	TSF
6	Mongrel	13 years 8 months	MN	48.0	TSF
7	Mongrel	3 years 9 months	FN	30.1	TSF
8	Mongrel	5 years 7 months	FN	28.0	PSRF
9	Weimaraner	1 year 2 months	M	38.5	PSRF
10	Bergamasco shepherd dog	6 years	MC	35.0	PSRF
11	Golden retriever	12 years 3 months	MC	28.0	PSRF
12	Dobermann	2 years 8 months	M	37.0	PSRF
13	Mongrel	8 years 2 months	F	35.0	PSRF
14	Mongrel	4 years 2 months	M	30.0	PSRF
15	Mongrel	2 years	M	25.2	SPMMA
16	Siberian husky	9 years 11 months	FN	38.0	SPMMA
17	Golden retriever	5 years 5 months	M	25.0	SPMMA
18	Labrador retriever	7 years 1 month	FN	28.7	SPMMA
19	German wirehaired pointer	10 years 6 months	MN	30.1	SPMMA
20	German shepherd	3 years 7 months	F	34.5	SPMMA
21	Boxer	10 years	MN	36.5	SPMMA

Abbreviations: F, female; FN, female neutered; M, male; MN, male neutered; PSRF, pedicle screw‐rod fixation; SPMMA, screws and polymethylmethacrylate; TSF, transarticular screw fixation.

### 2.1 Specimen preparation

All specimens were prepared and tested using a uniform protocol. As testing a single specimen required 24 h (see testing protocol below), specimens were processed sequentially. Each frozen specimen was thawed at 4°C for 24 h and cleared of all soft tissue except ligamentous structures and intervertebral discs. The L6–L7 intervertebral junction was stabilized by inserting a 3.5 mm stainless steel screw from L6 into L7 and a threaded 2.4 mm pin across both L6‐L7 facet joints. The cranial and caudal segment ends were then embedded in polymethylmethacrylate (PMMA; Suter Kunststoffe, Fraubrunnen, Switzerland) cylinders (diameters: 80 mm for L6, 60 mm for S3) after inserting two 3.5 mm screws into L6 and S3 to enhance PMMA–specimen interface stability. During preparation and testing, the specimens were kept moist by regularly spraying with saline solution (0.9% NaCl) and wrapping in saline‐soaked swabs.

### 2.2 Group assignment and conditions

Spinal specimens were randomly assigned to one of the following testing groups: (1) the TSF group, (2) the PSRF group, and (3) the SPMMA group. A sample size of *n* = 7 specimens per testing group was determined on the basis of previously published testing criteria for the standardization of in vitro stability testing of spinal implants.^13^

Each spinal specimen was tested in the following sequence:

1. Native spine (Condition 1).
2. Spine after surgical decompression and stabilization of L7–S1 (Condition 2).

For Condition 2, a dorsal laminectomy and partial discectomy of the L7–S1 intervertebral disc was performed to simulate a condition of spinal instability or degeneration. Subsequently, each specimen was surgically stabilized by one of the three methods, depending on group assignment.

#### 2.2.1 Transarticular screw fixation group

**FIGURE 1:**
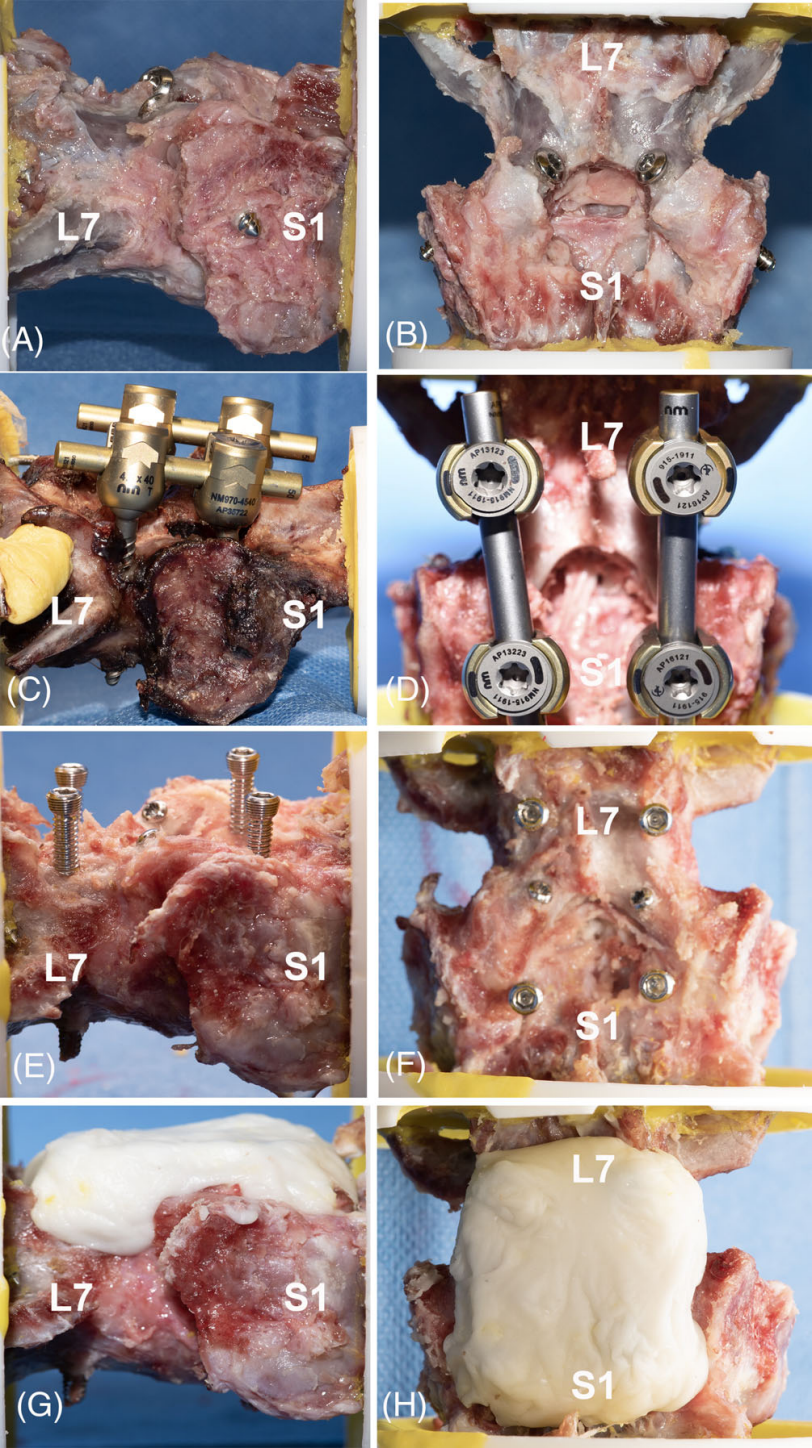
Lateral (left) and dorsal (right) views of canine lumbosacral spinal specimens stabilized using three techniques. (A, B) Transarticular screw fixation with 3.5 mm cortical screws. (C, D) Pedicle screw and rod fixation with 4.5 mm pedicle screws and a 5.5 mm rod (Neuromed). (E–H) Fixation with screws and polymethylmethacrylate (PMMA) using 2.4 mm transarticular cortical screws and 3.5 mm locking head screws: (E, F) without PMMA and (G, H) with PMMA applied.

The LSJ was stabilized as previously described.^8^, ^14^ Briefly, screw holes were drilled across the L7–S1 facets using a 2.5 mm drill bit and then tapped manually using a 3.5 mm cortical screw tap. The screw hole was directed ventrolaterally from the dorsomedial aspect of the cranial L7 articular process at an angle of 30–45° from the sagittal plane and 45–60° from the transverse plane, extending into the ventrolateral sacrum and seated in the body of S1. Screw size was determined by the width of the caudal L7 articular process, with a maximum screw diameter of 25% of this width to prevent fracture of the articular process.^15^ In each specimen, 3.5 mm cortical screws (Depuy Synthes, Johnson & Johnson, Oberdorf, Switzerland), were placed bilaterally (Figure 1A,B). Screw length varied from 26 to 40 mm, depending on specimen size and the insertion angle.

#### 2.2.2 Pedicle screw‐rod fixation group

The LSJ was stabilized using PSRF as previously reported.^5^, ^16^ Pedicle screw‐rod fixation employed 4.5 mm self‐tapping, polyaxial titanium pedicle screws interconnected with 5.5 mm titanium rods specifically designed for veterinary patients (Neuromed Invetra Pedicle Screw Fixation System, Orthomed, Huddersfield, UK; Figure 1C,D). Screws were placed bicortically into both pedicles of L7 and S1, with one thread protruding from the ventral vertebral cortex. Screw size was selected according to the manufacturer's recommendation, with the maximum diameter of the screws not exceeding 50% of the pedicle width.

#### 2.2.3 Screws and polymethylmethacrylate group

The LSJ was stabilized using transarticular cortical screws combined with locking head screws placed into the pedicles of L7 and S1. All screws were subsequently interconnected with PMMA as previously described.^9^, ^14^ Briefly, two 1.5 mm bone tunnels were created from the center of the caudal L7 articular process to the ipsilateral cranial sacral articular process, and 2 × 2.4 mm cortical screws (DePuy Synthes) were placed as transarticular screws. Screw corridors were then drilled bilaterally into the pedicles of L7 and S1 using a 2.5 mm drill bit, and 3.5 mm self‐tapping locking head screws (DePuy Synthes) were inserted bilaterally into the pedicles of L7 and S1 (Figure 1E,F). Thereafter, PMMA was applied to fill the shafts and heads of all screws (Figure 1G,H).

All surgical procedures were performed by a board‐certified veterinary surgeon (LAS).

### 2.3 Phase I: nondestructive biomechanical testing

The native spinal specimens (Condition 1) and specimens after surgical stabilization (Condition 2) were subjected to nondestructive biomechanical testing. Embedded specimens were mounted in a custom‐made six‐degree‐of‐freedom spine‐testing machine.^17^, ^18^ One potted end was fixed to an arm attached directly to a linear‐torsion all‐electric dynamic and static testing machine (Instron E3000, Instron Corporation IST), allowing control of bending motion. The other arm was attached to the spine‐testing machine.

Reflective markers were connected to the vertebral bodies of L7 and S1. Marker motion was recorded at 335 samples/s using an optical three‐dimensional (3D) pose tracking system with one array of two cameras (FusionTrack 500, Atracsys LLC, Puidoux, Switzerland). The system achieved a 3D precision of 0.08 mm (root mean squared error) at a camera‐to‐specimen distance of 2.0 m. Load and displacement data were sampled at 100 samples/s. Angular displacement (°) of the lumbosacral junction was calculated from the spatial coordinates of the markers.

Each specimen underwent the same biomechanical testing protocol: (1) flexion/extension (FE), (2) lateral bending (LB), and (3) axial rotation (AR). During FE and LB, specimens were deformed to their motion extremes at a constant rate of 0.3°/s and subjected to a bending moment of ±3 Nm applied across L7–S1. Axial compression equal to 60% of body weight was applied to all specimens.^19^ For AR, torsional load was applied using displacement‐controlled movement at 0.1°/s. Each specimen underwent three loading cycles per motion direction.

### 2.4 Phase II: fatigue cyclic testing

Each stabilized spinal specimen (Condition 2) underwent biomechanical fatigue testing. Specimens were subjected to 200 000 cycles of FE at a constant bending moment of ±2 Nm at a loading speed of 5 Hz. Range of motion (ROM;°) was recorded continuously throughout the 200 k cycles. Construct failure was defined as ROM reaching 5°. The testing software automatically tracked ROM, and testing was stopped immediately if ROM exceeded 5° before completing 200 000 cycles. Cycles to failure were recorded for each specimen.

### 2.5 Phase III: ramped cyclic testing

Specimens that did not fail after 200 000 cycles underwent ramped cyclic testing. Each specimen was incrementally loaded from 2 Nm to 10 Nm in 0.5 Nm steps. At each bending moment, 100 cycles of FE were applied before increasing the load. Testing continued until either 100 loading cycles at 10 Nm were completed or specimen failure occurred.

### 2.6 Failure modes

For both fatigue and ramped cyclic testing, construct failure was defined as a ROM of 5°. Following testing, specimens were inspected for the following failure modes:

- Fracture of the bone specimen
- Fracture of an implant
- Screw loosening, defined as angular or linear displacement of the screw relative to the vertebra, with halo formation at the screw–bone interface

Failure modes were assessed visually during and after testing and from the load–displacement data. Bone or implant fracture appeared on the load–displacement curve as a sudden loss of stiffness or abrupt increase in ROM, whereas screw loosening was characterized by a gradual, progressive increase in ROM.

### 2.7 Data analysis and parameters

For nondestructive biomechanical testing, only data for the third loading cycle of each motion series were used. The following parameters were determined for Condition 1 and 2 in each specimen:

- ROM: the range of angular displacement between the minimum (−3 Nm) and maximum (+3 Nm) applied moments.
- Neutral zone (NZ): the range of angular displacement within which the specimen moved with minimal applied load, calculated between −0.1 Nm and +0.1 Nm of applied moment.
- Neutral zone stiffness (NZS): the quotient of loading and angular displacement in the NZ. This was calculated from the upward slope of the load–displacement curve.^13^

The ROM, NZ, and NZS were calculated for FE, LB, and AR.

- For fatigue cyclic testing, the ROM (°) of the LSJ was recorded after 50 000, 100 000, 150 000, and 200 000 cycles. If a specimen failed (i.e., ROM > 5°), the number of cycles to failure and failure mode were recorded.
- For ramped cyclic testing, the ROM (°) at 4 Nm through 10 Nm was recorded. For each specimen, the bending moment and number of cycles corresponding to 5°, 7.5°, and 10° of ROM were recorded. These absolute ROM values (5°, 7.5°, and 10°) were selected as limits for increased mobility or progressive construct failure because none of the stabilized constructs achieved absolute stability (i.e., ROM of 0°) immediately after stabilization; therefore, no single fixed ROM limit for failure could be defined. The failure mode associated with progressive mobility was recorded for each specimen.

### 2.8 Statistics

For the continuous data, means ± SD were calculated for all parameters. For descriptive statistics, bar graphs were generated for each parameter. Statistical analyses were performed using R statistical software.^20^ Linear mixed models, containing both fixed and random effects,^21^ were used to analyze the continuous parameters for nondestructive biomechanical testing, fatigue cyclic testing, and ramped fatigue testing. All models were assessed for the following conditions: (1) Relationship between fixed effects and response (linearity); (2) homoscedasticity; (3) normal distribution of residuals; (4) normal distribution of random effects; and 5) multicollinearity. For some parameters, a log transformation was used to improve these conditions. For all models/parameters, random effects included in the model were *“*spine*”* (1–21). For nondestructive testing, fixed effects were *“*condition*”* (native specimen, stabilized specimen), *“*motion direction*”* (FE, LB, AR), and *“*method of stabilization*”* (TSF, PSRF, SPMMA). For cyclic fatigue testing, the fixed effects included *“*method of stabilization*”* (TSF, PSRF, SPMMA) and *“*number of loading cycles*”* (50 000, 100 000, 150 000, 200 000). For ramped cyclic testing, the fixed effects included *“*method of stabilization*”* (TSF, PSRF, SPMMA) and ROM limits (5°, 7.5° and 10° of ROM). Fisher's exact test was used to assess differences in failure modes between TSF, PSRF, and SPMMA.

The Benjamini–Hochberg adjustment for multiple comparisons was applied. All *p* values generated from the hypothesis tests were collected and ranked from smallest to largest; *p* values were corrected using a correction factor calculated on the basis of their rank:

$$\left(m/i\right)\cdot p$$

where *m* = total number of tests; *i* = rank; and *p* = the original *p* value. Multiplicity‐adjusted *p* values were generated using this methodology. Multiplicity‐adjusted *p* < .05 was considered statistically significant.

## 3 RESULTS

### 3.1 Nondestructive biomechanical testing

**FIGURE 2:**
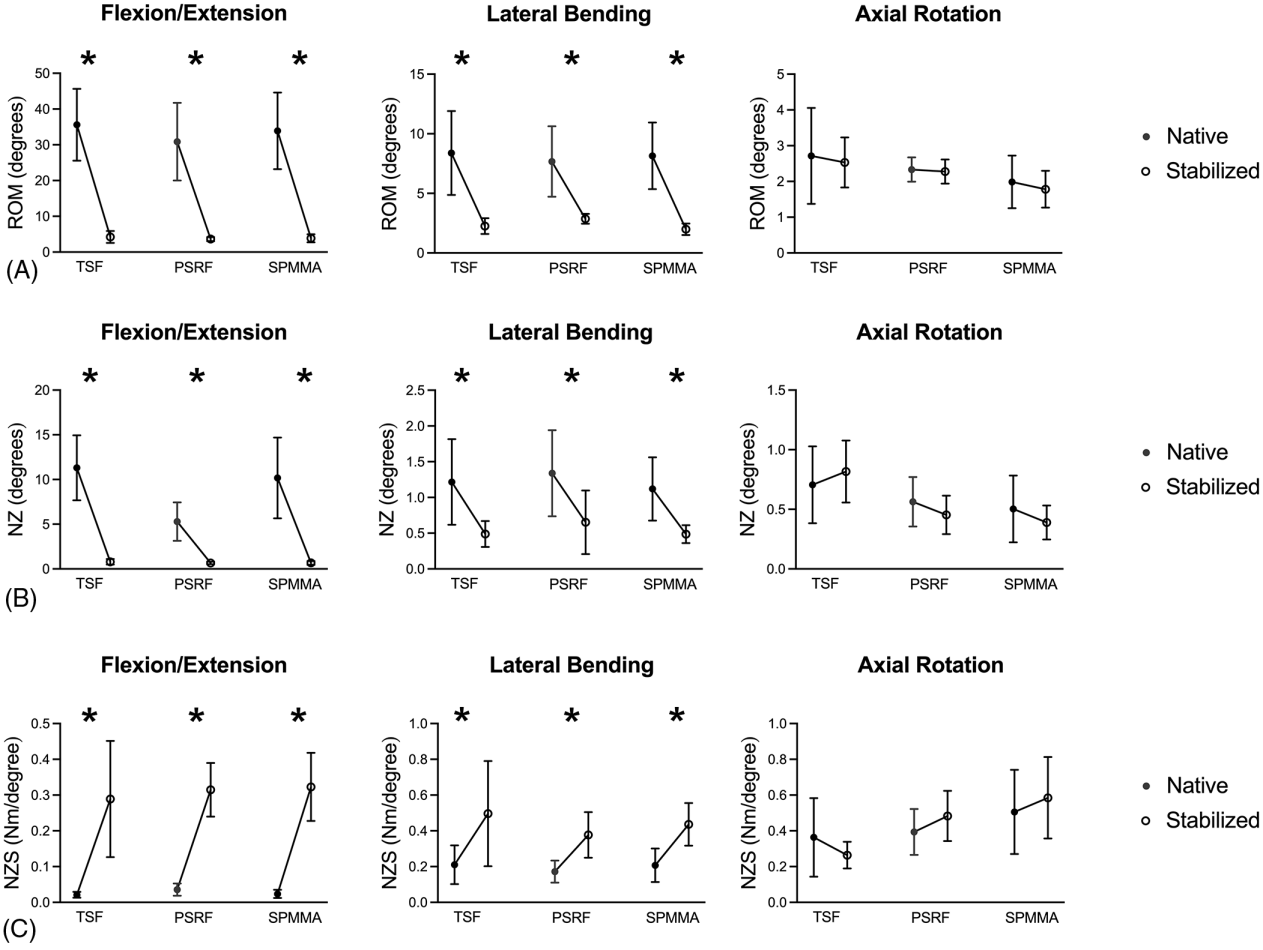
Line graphs (mean ± SD) showing (A) range of motion (ROM), (B) neutral zone (NZ), and (C) neutral zone stiffness (NZS) in flexion–extension, lateral bending, and axial rotation for the native spine and after stabilization with transarticular screw fixation (TSF), pedicle screw and rod fixation (PSRF), and screws and polymethylmethacrylate (SPMMA) fixation. An asterisk (*) indicates a significant difference (*p* < .05) between the native and stabilized conditions within each group.

Range of motion (ROM): All three methods of L7‐S1 stabilization resulted in a decrease (mean ± SD across all stabilization groups) in ROM in FE (−29.6 ± 10.0°; *p* < 0.01) and LB (−5.7 ± 2.8°, *p* < .01), but not in AR (−0.2 ± 0.7°, *p* = .47; Figure 2A; Table 2 and Supporting Information, Table S1). The ROM after L7‐S1 stabilization did not differ among TSF, PSRF, and SPMMA in FE (3.9 ± 1.2°, *p* = .74), LB (2.4 ± 0.6°, *p* = .98), and AR (2.2 ± 0.6°, *p* = .20).

**TABLE 2:** Means ± SDs for range of motion (ROM), neutral zone (NZ), and neutral zone stiffness (NZS) in flexion/extension, lateral bending, and axial rotation for the stabilization groups: transarticular screw fixation (TSF), pedicle screw–rod fixation (PSRF), and screw and polymethylmethacrylate fixation (SPMMA)

	ROM (°)		NZ (°)		NZS (Nm/°)	
	N	S	N	S	N	S
	*Flexion/extension*					
TSF	35.6 ± 10.0	4.2 ± 1.7	11.3 ± 3.6	0.8 ± 0.3	0.02 ± 0.01	0.29 ± 0.16
PSRF	30.9 ± 10.9	3.6 ± 0.6	5.3 ± 2.1	0.7 ± 0.1	0.04 ± 0.02	0.31 ± 0.07
SPMMA	33.9 ± 10.7	3.9 ± 1.1	10.2 ± 4.5	0.7 ± 0.2	0.02 ± 0.01	0.32 ± 0.10
	*Lateral bending*					
TSF	8.4 ± 3.5	2.3 ± 0.7	1.2 ± 0.6	0.5 ± 0.2	0.21 ± 0.11	0.49 ± 0.29
PSRF	7.7 ± 3.0	2.9 ± 0.4	1.4 ± 0.6	0.6 ± 0.4	0.17 ± 0.06	0.38 ± 0.13
SPMMA	8.1 ± 2.8	2.0 ± 0.5	1.2 ± 0.4	0.5 ± 0.1	0.21 ± 0.09	0.44 ± 0.12
	*Axial rotation*					
TSF	2.7 ± 1.3	2.5 ± 0.7	0.7 ± 0.3	0.8 ± 0.3	0.36 ± 0.22	0.26 ± 0.07
PSRF	2.3 ± 0.3	2.3 ± 0.4	0.6 ± 0.2	0.5 ± 0.2	0.39 ± 0.13	0.48 ± 0.14
SPMMA	2.0 ± 0.7	1.8 ± 0.5	0.5 ± 0.3	0.4 ± 0.1	0.50 ± 0.23	0.58 ± 0.23

Abbreviations: N, native; S, stabilized.

Neutral zone (NZ): All three methods of L7‐S1 stabilization resulted in a decrease in NZ in FE (−8.2 ± 4.3°; *p* < .01) and LB (−0.7 ± 0.5°; *p* < .01), but not in AR (−0.04 ± 0.3°, *p* = .73; Figure 2B). The NZ after L7‐S1 stabilization did not differ between TSF, PSRF, and SPMMA in FE (0.71 ± 0.23°, *p* > .46), LB (0.54 ± 0.28°, *p* = .71), and AR (0.55 ± 0.27°, *p* = .34).

Neutral zone stiffness (NZS): All three stabilization methods resulted in an increase in NZS in FE (+0.28 ± 0.11 Nm/°; *p* < .01) and LB (+0.24 ± 0.18 Nm/°; *p* < .01), but not in AR (+0.03 ± 0.21 Nm/°, *p* = .73; Figure 2C). The NZS after L7‐S1 stabilization did not differ between TSF, PSRF, and SPMMA in FE (0.31 ± 0.11 Nm/°, *p* = .17), LB (0.44 ± 0.19 Nm/°, *p* = .66), and AR (0.44 ± 0.20°, *p* = .20).

### 3.2 Fatigue cyclic testing

**FIGURE 3:**
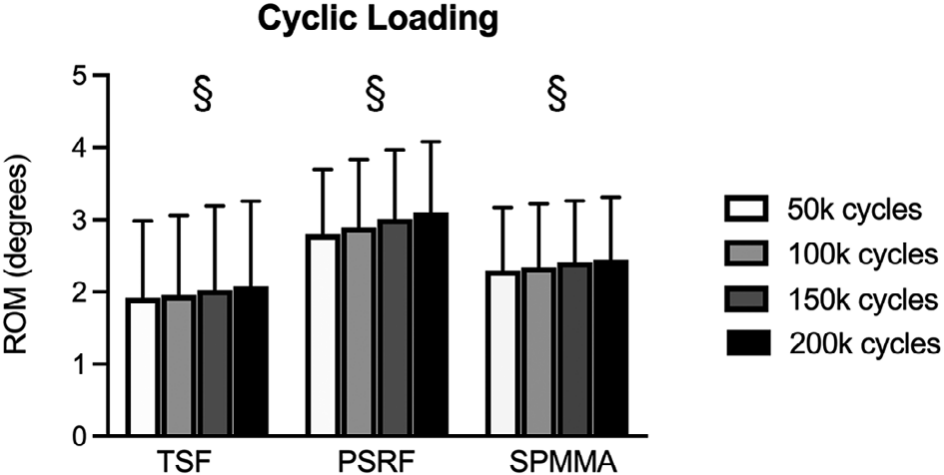
Range of motion (ROM; mean ± SD) in flexion–extension of lumbosacral spinal specimens stabilized with transarticular screw fixation (TSF), pedicle screw and rod fixation (PSRF), and screws with PMMA (SPMMA) during fatigue cyclic testing. The section mark (§) indicates an increase in ROM from 50 000 to 200 000 cycles. Although *p* < .05, these changes were minor.

Fatigue cyclic testing resulted in progressive, but minor increases in ROM compared to baseline (Figure 3). Under cyclic testing, the ROM progressively increased to 2.34 ± 0.97°, 2.40 ± 1.10°, 2.48 ± 1.10°, 2.54 ± 1.05° (*p* < .01) at 50 000, 100 000, 150 000, and 200 000 loading cycles, respectively (Supporting Information, Table S1). No differences were found between TSF, PSRF, and SPMMA at 50 000, 100 000, 150 000, and 200 000 loading cycles (*p* = .31). Fatigue cyclic testing up to 200 000 loading cycles of FE did not result in failure (defined as ROM > 5.0°) in any of the specimens. No implant failure was observed under cyclic testing for any of the specimens.

### 3.3 Ramped cyclic testing

**FIGURE 4:**
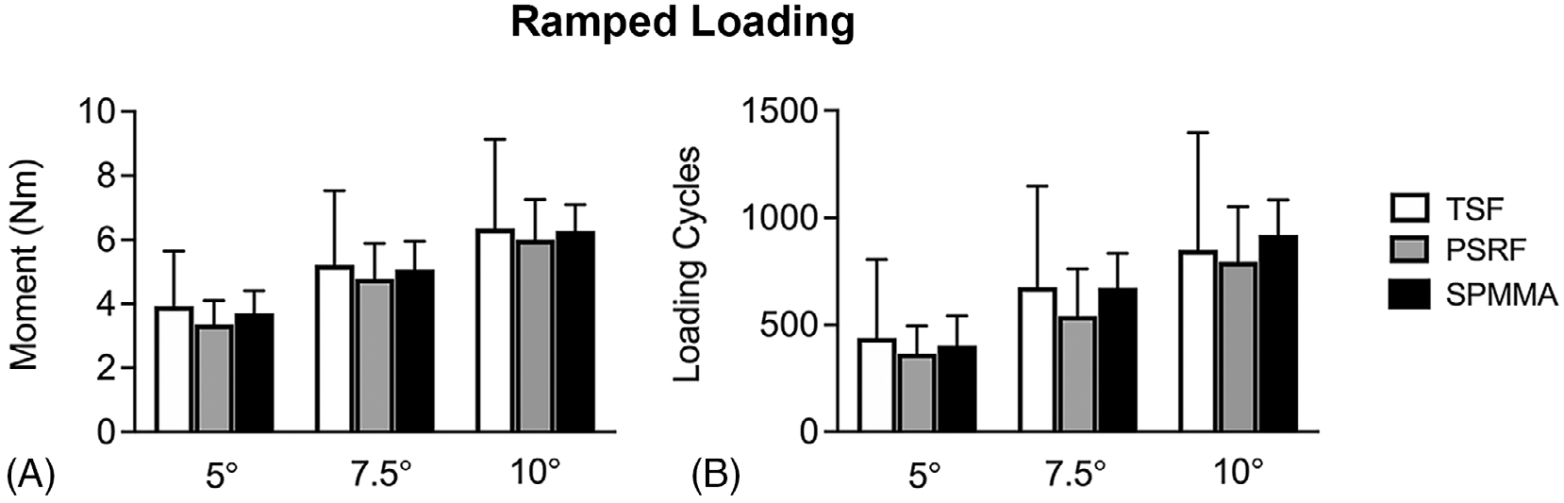
Mean ± SD for (A) bending moment (Nm) and (B) number of loading cycles required to reach 5°, 7.5°, and 10° of range of motion (ROM) for the transarticular screw fixation (TSF), pedicle screw and rod fixation (PSRF) and screws and (SPMMA) groups during ramped cyclic testing.

All stabilization groups showed a progressive increase in ROM and, hence, progressive failure when subjected to ramped cyclic testing (*p* < .01; Figure 4). No differences were found between TSF, PSRF, and SPMMA with respect to the bending moment necessary to reach 5.0°, 7.5° and 10.0° of ROM (*p* = .91). Similarly, no differences were found among the testing groups with respect to the numbers of loading cycles necessary to reach 5°, 7.5°, and 10.0° of ROM (*p* = .91).

### 3.4 Failure modes

All constructs showed progressive failure during ramped cyclic testing through one of three modes: (1) screw loosening, (2) fracture of L7, and (3) fracture through the sacrum (Table 3). No differences with respect to mode of failure were found among TSF, PSRF, and SPMMA (*p* > .67).

**TABLE 3:** Overview of failure modes for the transarticular screw group (TSF), pedicle screw–rod fixation group (PSRF), and screws and polymethylmethacrylate group (SPMMA).

	*Screw loosening*	*L7 fracture*	*Sacrum fracture*
TSF	6/7 (85.7%)	1/7 (14.3%)	1/7 (14.3%)
PSRF	5/7 (71.4%)	0/7 (0%)	2/7 (28.6%)
SPMMA	4/7 (57.1%)	0/7 (0%)	3/7 (42.9%)

For the TSF group, the main mode of failure was screw loosening (6/7 specimens, 85.7%), resulting in progressive screw pullout from S1 and the L7 articular process. In one of these specimens, progressive screw loosening of one screw was followed by a fracture through S1 on the contralateral side. One specimen failed through fracture of the L7 articular process (1/7 specimens, 14.3%).

For the PSRF group, the main mode of failure was bilateral screw loosening of the S1 screws (5/7 specimens; 71.4%). This screw loosening mainly involved loss of bone stock surrounding the screws as a result of repeated loading. The other mode of failure involved fracture of the sacrum (2/7; 28.6%). Fracture of the pedicle screws and connecting rods was not observed in any of the specimens.

For the SPMMA group, the main mode of failure was bilateral screw loosening of the S1 screws (4/7 specimens; 57.1%). This screw loosening mainly involved loss of bone stock surrounding the S1 pedicle screws as a result of repeated loading. Other modes of failure included fracture of the sacrum (3/7; 42.9%). Fracture of the screws and PMMA was not observed in any of the specimens.

## 4 DISCUSSION

This study provides insights into the biomechanical performance of three surgical stabilization techniques for DLSS in dogs. The first hypothesis, that all three stabilization methods would provide direct postoperative rigid fixation of the LSJ, was supported by the decrease in ROM and NZ, and the increase in NZS following stabilization. However, the second hypothesis, which posited that PSRF and SPMMA would be superior to TSF in withstanding fatigue cyclic testing and ramped cyclic testing, was rejected, because performance did not differ among the techniques. The third hypothesis, which suggested that the three stabilization methods would exhibit different modes of failure, was rejected. All three methods mainly showed progressive screw loosening during ramped cyclic testing; in the TSF group, because the screws were not contained in a fixed construct, screw loosening was associated with progressive screw pull‐out. In the PSRF and SPMMA groups, ramped testing resulted in progressive loosening of the S1 screws; in some instances, fracture of the sacrum was observed.

### 4.1 Immediate Stability

This ex vivo study provides the first biomechanical analysis comparing three commonly used surgical techniques for stabilization of the canine LSJ. The ROM, NZ, and NZS parameters were analyzed to assess immediate postoperative stability.

In spinal biomechanics, the ROM represents the total angular displacement that a motion segment can achieve under loading. However, it does not describe how motion behaves within that range. The ROM can be subdivided into the NZ and the elastic zone.^13^ The NZ quantifies motion around the neutral position where the spine moves with minimal internal resistance—the “slack” before ligaments, facets, or implants tighten. It is a useful parameter in biomechanical studies because it reflects control and stiffness around the physiological resting position. As most physiological loads and daily activities occur near the neutral posture rather than at extreme ranges, NZ complements ROM by identifying subtle changes in laxity and control, thereby indicating the functional effectiveness of spinal stabilization near the neutral position.

All three methods stabilized the LSJ in FE and LB. These results align with previous biomechanical studies on LS stabilization using PSRF.^16^, ^22^ However, as far as the authors are aware, this is the first study to evaluate the biomechanical effects of TSF and SPMMA constructs specifically at L7–S1.

In contrast with FE and LB, none of the stabilization methods was shown to reduce ROM, NZ, and NZS in axial rotation. This is likely attributable to the small ROM and NZ in axial rotation—only a few degrees—in comparison with FE and LB (Table 2). Previous ex vivo biomechanical studies have shown that the canine lumbosacral functional spinal unit exhibits relatively low ROM and NZ in axial rotation relative to other motion directions.^6^, ^25^ Consequently, detecting changes in ROM or NZ for axial rotation is inherently difficult due to the substantial stiffness characteristic of this mode of motion.

### 4.2 Biomechanical Fatigue Testing

The experimental design in this study incorporated a combination of fatigue cyclic and ramped cyclic testing. For cyclic loading, 200 000 cycles were applied to simulate controlled testing over a 12‐week postoperative period. This value was based on recent fluoroscopic studies indicating that walking at 1.3 m/s corresponds to approximately 40 gait cycles per minute.^23^ Assuming a controlled daily exercise regimen of 45 minutes on a leash, this equates to approximately 1800 gait cycles per day. Consequently, 200 000 cycles approximate 111 days of exercise, representing a 3 month postoperative period. Transarticular screw fixation, PSRF, and SPMMA stabilization were able to withstand the complete cyclic testing protocol of 200 000 cycles, showing only minor increases in total ROM in flexion/extension. None of the constructs was observed to fail during the fatigue cyclic testing phase.

None of the constructs failed under cyclic testing so a ramped cyclic testing protocol was subsequently applied. Unlike conventional “load‐to‐failure” testing, which involves a linear increase in bending moment until catastrophic failure occurs, the ramped protocol was selected to better simulate fatigue‐related failure, which may be more representative of in vivo conditions. The TSF, PSRF, and SPMMA all showed a progressive loss in stability when subjected to ramped cyclic testing, which mainly resulted from progressive screw loosening in all groups. The weak link for all three groups appeared to be the sacrum, which consists of relatively soft bone relative to L7.

Contrary to the study's hypothesis, TSF demonstrated fatigue resistance comparable to that of PSRF and SPMMA. It may thus be concluded that these methods of fixation offer equivalent clinical stability, provided the implants are placed appropriately.

Critical factors for the effectiveness of TSF are the screw insertion angle and screw length used. Optimal screw insertion requires a caudal and lateral orientation to maximize sacral engagement, as inadequate engagement can substantially compromise the stability of the construct. Screws that are inserted with an excessively steep ventral angle may fail to adequately anchor in the sacrum, leading to increased risk of loosening and eventual stabilization failure. Similarly, insufficient screw length may prevent adequate penetration into S1, diminishing resistance to physiological loads.

As this study used cadaveric specimens, transarticular screws were placed under direct visual control, allowing ideal angulation and length, with the screw tips just penetrating the lateral border of S1. This optimal placement may explain the considerable resistance to failure observed in the present study. In clinical settings, however, suboptimal screw angulation or length may occur, leading to screw loosening and pull‐out in a substantial proportion of cases as previously reported (23.5%).^8^ Clinicians employing TSF should therefore adjust screw trajectory and length carefully to minimize the risk of stabilization failure.

Pedicle screw‐rod fixation and SPMMA performed in a very similar way with respect to biomechanical fatigue testing. The main complications reported for PSRF in a clinical scenario include cortical encroachment or frank penetration of the spinal canal when placing the pedicle screws. Implant failure, involving fracture of either the L7 or S1 screws, has not been reported so far.^12^, ^24^ In contrast, SPMMA fixation has been associated with screw fractures of the L7, S1 and transarticular screws, occurring between 3 and 12 months postoperatively.^9^ The absence of reported implant failure in PSRF compared to SPMMA fixation may be attributed to the selected screw sizes: reports on SPMMA used 3.5 mm locking head screws,^9^ whereas reports on PSRF consistently used screws with a minimum diameter of 4.0 mm.^5^, ^6^, ^12^, ^22^, ^24^ Selecting pedicle screws with a larger diameter might therefore help to mitigate implant failure. In this study, no screw fractures were observed, likely due to the controlled nature of the experimental design. The LS specimens were subjected to fatigue cyclic and ramped cyclic testing in FE, whereas in clinical scenarios, the LSJ might experience sudden, high‐impact movements involving flexion, rotation, and compression, which increase the risk of implant failure. However, based on the results of the present work, it might be hypothesized that progressive loosening of the S1 screws, which have relatively short screw corridors compared to L7 screws, could lead to undesired micromotion of the screws, predisposing them to subsequent fracture.

### 4.3 Limitations

The limitations of this study must be acknowledged. These are primarily due to its ex vivo design. Although failure testing was conducted in FE, recent kinematic evidence suggests that other movements, such as axial rotation, may play a more important role in the biomechanical behavior of the LSJ during physiological activity. Flexion‐extension was selected as the primary movement for analysis because it exhibits the greatest ROM and NZ, as well as the least stiffness within the LS spine, thereby necessitating stabilization. However, understanding in vivo motion dynamics remains critical, as activities such as sitting and jumping may also contribute to implant failure. Future studies should incorporate a more comprehensive evaluation of multiple (coupled) motion patterns, ideally assessing specimens under loading conditions in all relevant directions until failure occurs.

Biomechanical fatigue testing was conducted over a total duration of 24 hours per specimen. Although care was taken to process the specimens uniformly and preserve specimen integrity and hydration, some loss of biomechanical integrity from gradual tissue decomposition cannot be excluded.

### 4.4 Conclusion

In conclusion, TSF, PSRF, and SPMMA provided immediate biomechanical stability to the LSJ and exhibited comparable resistance to cyclic testing over 200 000 cycles in FE. However, ramped cyclic testing resulted in a progressive loss of stability across all constructs, with no single method demonstrating clear superiority. Based on these findings, all three techniques can be considered effective and reliable options for stabilizing the canine LSJ.

## AUTHOR CONTRIBUTIONS

Smolders LA, PhD, DECVS: Designed the study, collected the cadaveric specimens, performed the surgery and the experiments, performed data collection and analysis, drafted and finalized the manuscript. Arz R, Dr med vet: Contributed to the design of the study, assisted in the surgical procedures, performed the experiments, and edited the manuscript. Steffen F, DECVN: Contributed to the study design, participated in data interpretation, and edited the manuscript. Pozzi A, MS, DACVS (Small Animal), DECVS, DACVSMR: Contributed to the study design, participated in data interpretation, and edited the manuscript. Park B, PhD: Contributed to the study design, supervised the biomechanical experiments, participated in and supervised data analysis and interpretation, and edited the manuscript. All authors provided a critical review of the manuscript and endorse the final version. All authors are aware of their respective contributions and have confidence in the integrity of all contributions.

## CONFLICT OF INTEREST

This study was supported by Orthomed (Huddersfield, UK), which supplied the implants for PSRF free of charge. No other conflicts of interest are reported.

## Data Availability

The raw biomechanical data from the study are available.
